# A novel glycoside hydrolase 43-like enzyme from *Clostridium boliviensis* is an endo-xylanase and a candidate for xylooligosaccharide production from different xylan substrates

**DOI:** 10.1128/aem.02223-23

**Published:** 2024-03-18

**Authors:** Daniel Martin Salas-Veizaga, Leonardo Roberto Rocabado-Villegas, Javier A. Linares-Pastén, Elisabet Eik Gudmundsdottir, Gudmundur Oli Hreggvidsson, María Teresa Álvarez-Aliaga, Patrick Adlercreutz, Eva Nordberg Karlsson

**Affiliations:** 1 Division of Biotechnology, Department of Chemistry, Lund University, Lund, Sweden; 2 Instituto de Investigaciones Fármaco Bioquímicas, Universidad Mayor de San Andrés, La Paz, Bolivia; 3 Matis Ohf, Reykjavik, Iceland; University of Milano-Bicocca, Milan, Italy

**Keywords:** *Clostridium boliviensis* strain E-1, endo-β-xylanase, GH43-L, xylooligosaccharides, quinoa stalks glucuronoarabinoxylan, wheat arabinoxylan, birchwood xylan

## Abstract

An uncharacterized gene encoding a glycoside hydrolase family 43-like enzyme from *Clostridium boliviensis* strain E-1 was identified from genomic sequence data, and the encoded enzyme, *CbE1*Xyn43-l, was produced in *Escherichia coli. CbE1*Xyn43-l (52.9 kDa) is a two-domain endo-β-xylanase consisting of a C-terminal CBM6 and a GH43-like catalytic domain. The positions of the catalytic dyad conserved in GH43, the catalytic base (Asp74), and proton donor (Glu240) were identified in alignments including GH43-enzymes of known 3D-structure from different subfamilies. *CbE1*Xyn43-l is active at pH 7.0–9.0, with optimum temperature at 65°C, and a more than 7 days’ half-life in irreversible deactivation studies at this temperature. The enzyme hydrolyzed birchwood xylan, quinoa stalks glucuronoarabinoxylan, and wheat arabinoxylan with xylotriose and xylotetraose as major hydrolysis products. *CbE1*Xyn43-l also released xylobiose from *p*NPX_2_ with low turnover (*k*_cat_ of 0.044 s^−1^) but was inactive on *p*NPX, showing that a degree of polymerization of three (DP3) was the smallest hydrolyzable substrate. Divalent ions affected the specific activity on xylan substrates, which dependent on the ion could be increased or decreased. In conclusion, *CbE1*Xyn43-l from *C. boliviensis* strain E-1 is the first characterized member of a large group of homologous hypothetical proteins annotated as GH43-like and is a thermostable endo-xylanase, producing xylooligosaccharides of high DP (xylotriose and xylotetraose) producer.

**IMPORTANCE:**

The genome of *Clostridium boliviensis* strain E-1 encodes a number of hypothetical enzymes, annotated as glycoside hydrolase-like but not classified in the Carbohydrate Active Enzyme Database (CAZy). A novel thermostable GH43-like enzyme is here characterized as an endo-β-xylanase of interest in the production of prebiotic xylooligosaccharides (XOs) from different xylan sources. *CbE1*Xyn43-l is a two-domain enzyme composed of a catalytic GH43-l domain and a CBM6 domain, producing xylotriose as main XO product. The enzyme has homologs in many related *Clostridium* strains which may indicate a similar function and be a previously unknown type of endo-xylanase in this evolutionary lineage of microorganisms.

## INTRODUCTION

With the increasing demand to use renewable resources for the production of various types of food additives and platform of chemicals, interest in the isolation of novel enzymes acting on hemicellulose (a major part of renewable biomass) has increased to find candidate enzymes that act selectively on these polymers. Xylans are the most common polymers in hemicellulose from many types of complex biomass, and in most cases, xylans are underutilized industrially. There is, thus, considerable potential in obtaining xylans from industrial side streams and upgrading them to valuable products, like prebiotic oligosaccharides (1). Examples of suitable side streams include those from the processing of different types of agricultural materials in the food and bio-energy industries.

The most well-known enzyme types acting on xylans are the endo-xylanases (EC 3.2.1.8), which are most commonly classified under glycoside hydrolase family 10 and 11 (GH10 and 11). In addition, arabinoxylanases (requiring arabinose substituents) have also been identified in GH5, and xylanases requiring glucuronosylation have been identified in GH30 (1). The GH-families 5, 10, 11, and 30 are all examples of enzymes with a retaining mechanism (2).

A few enzymes with endo-acting xylanase activity have been reported in inverting GH families, including a few examples in GH43 (3, 4). Glycoside Hydrolase family 43 is a large GH family that consists of more than 36,000 genes (www.cazy.org, accessed 2023-11-11) and is grouped into 38 subfamilies (5). The major activities described for this family include β-xylosidases, α-l-arabinofuranosidases, arabinanases, and β-galactosidases (6). The first 3D-structure of a catalytic domain from GH43 was described by Nurizzo et al. (7) and was an arabinanase now classified under GH43 subfamily 5 (6). Since then, enzyme candidates in a number of additional subfamilies have been structure determined. Structure-determined GH43 enzymes, in all subfamilies, have been shown to display a five-bladed β-propeller fold with a conserved dyad of amino acid residues in the active site, one Asp and one Glu (8).

In addition to the enzymes classified under this family, there is also a large number of genes deposited in databases (e.g., Genbank) that are annotated as GH43-like by the Conserved Domains Database (CDD) (https://www.ncbi.nlm.nih.gov/Structure/cdd/cdd.shtml). These annotated GH43-like genes belong to Clan F together with other conserved GH43_62_32_68_117_130-like domains curated by NCBI (cd08994). However, the sequential and strutural analysis show a higher sequence similarity of these genes to GH43 family enzymes than to the others. Nevertheless, despite to this group of hypothetical enzymes are more similar to GH43 than others among the glycoside hydrolase families, they still have limited sequences similarity to GH43 family as well, and are not yet present in the Carbohydrate Active Enzyme Database (CAZy, at www.cazy.org). No activity profiles are, to our knowledge, reported from the GH43-like enzymes, and no 3D-structures have yet been published from this subgroup of enzymes.

Enzymes in several of the GH43 enzyme subfamilies are reported to act on xylan. Most of these enzymes are exo-acting, including β-xylosidases (EC 3.2.1.37) acting on the xylan backbone, or arabinofuranosidases (EC 3.2.1.55) acting on the arabinosyl substituents attached to the xylan backbone (9), hydrolyzing α(1, 2) (10), α(1–3) (11) or double α (1, 2) and α (1–3) arabinofuranosyl bonds (12). Nonetheless, no GH43-like enzyme has yet been reported to have activity.

As stated above, a few enzymes in GH43 are also reported to display endo-xylanase activity (EC 3.2.1.8) (www.cazy.org). Applications for endo-xylanases include the production of oligosaccharides from different types of xylans. The most studied and described endo-xylanases belong to families GH10 and GH11 (13). However, when substituted xylose-based hemicelluloses (e.g., quinoa stalks glucuronoarabinoxylan) were hydrolyzed by GH10 and GH11 enzymes, this resulted in a low production of linear XOs (14).

Hence, to search for novel enzymes, the xylan-utilizing bacterium *Clostridium boliviensis* strain E-1, previously isolated in Bolivia (15), was genome sequenced, and the draft genome was searched for genes encoding hemicellulose active enzymes candidates. A number of at least 12 genes encoding potential hemicellulose active enzymes were identified, and a gene with significant sequence identity to genes annotated as GH43-like was cloned and the recombinant was produced in *E. coli*. To our knowledge, this is the first enzyme to be characterized from this large group of homologs. It is a two-domain endo-xylanase, comprising an N-terminal catalytic domain connected to Carbohydrate Binding Module from family 6 (CBM6). The enzyme was shown to produce XOs from three different types of xylans: xylan from birchwood, wheat arabinoxylan, and quinoa stalks glucuronoarabinoxylan.

## RESULTS

### Draft genome sequence of *Clostridium boliviensis* strain E-1 and genes encoding putative xylanolytic enzymes

*C. boliviensis* strain E-1 is a bacterium discovered in a bolivian region characterized by high altitude (>4,000 m.a.s.l), high UV-radiation, and temperature variation between day and night of about 20°C (15). Initially investigated as potential sulfate-reducing bacteria, *C. boliviensis* E-1 also showed the capability of growing in complex carbohydrate substrates (such as xylan), making it suitable for investigation from a biorefinery point of view, taking into account that the degradation of complex carbohydrates is directly related to the presence of enzymes with the capability of hydrolyzing them inside its genome.

A first draft genome was assembled from the sequencing of two libraries, made with two sequencing library methods and from two different DNA extraction methods. This draft genome was a total of 5.2 Mbp in 152 contigs over 500 bp. With the addition of a third sequencing library made by the Mate Pair method, the total genome size was increased to 5.5 Mbp and a number of contigs (over 500 bp) increased to 542; however, the N50 contig size increased from 216,596 to 753,199 bp and the largest contig from 446,924 to 2,435,166 bp, both indicated an improved assembly of a large fraction of the genome.

### GH43-like candidate identification

Annotation of the genes revealed a total of 12 genes encoding enzymes with putative xylan degrading activity or xylan-binding ability (data not shown), being all the candidates located by orthology in the family GH43 of glycoside hydrolases. A gene encoding a putative two-domain enzyme (here named *CbE1*Xyn43-l) was selected for further sequence analysis, cloning, production, and characterization. However, when the selected candidate was aligned with a representative member of each of the subfamilies belonging to the GH43 family, showed a sequence homology of 27.5% to the representative of subfamily GH43_25, 36.6% to GH43_15, 60.6% to GH43_16, and 74.3% to the representative of subfamily GH43_10; all representatives sequences from the other subfamilies had a sequence homology of less than 20%. These data indicate that the identified enzyme (*CbE1*Xyn43-l) shares a common ancestor with the GH43 family; however, it is possible that it belongs to an unclassified subfamily. On the other hand, the data showed significant sequence similarities with reported GH43-like sequences (up 80%), and it was confirmed by conserved domain analysis.

### Sequence comparison and modeling of the 3D-structure

**Fig 1:**
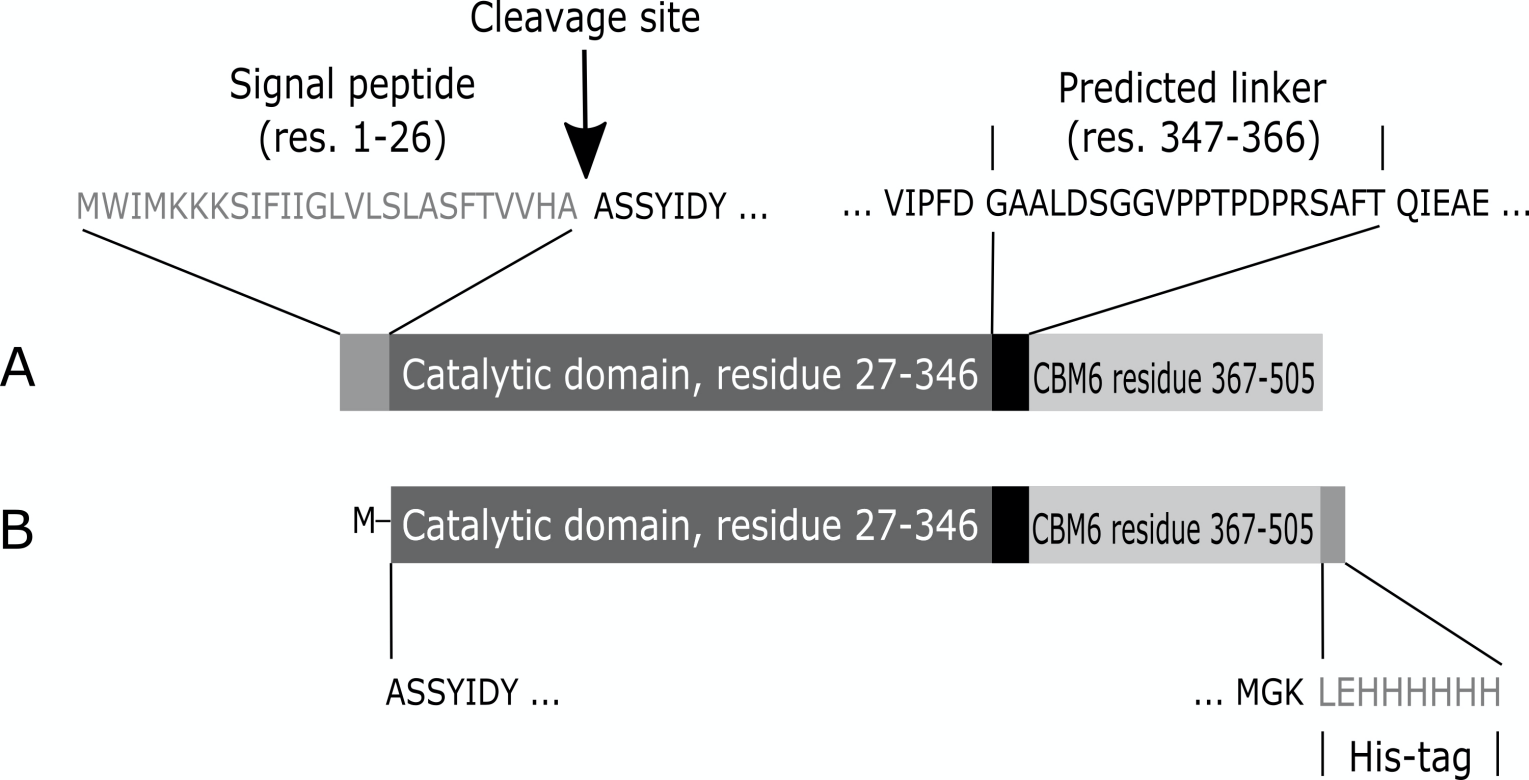
Block diagram showing the domain structure of the *Cb*E1Xyn43-l enzyme. (A) Native enzyme including the 26 residues signal peptide, the 320 residues catalytic domain followed by the predicted linker (20 residues), and the C-terminal family 6 Carbohydrate Binding Module (CBM6, 139 residues). (B) The amino acid sequence of the recombinant produced *Cb*E1Xyn43-l. The signal peptide is removed and replaced by a Met start codon. A six-residue His-tag is added to the C-terminus.

Based on the sequence similarities, *CbE1*Xyn43-l seems to be a two-domain protein with an N-terminal GH43-like putative catalytic domain of 320 residues (Ala27 to Asp346) and a C-terminal family 6 Carbohydrate Binding Module of 139 residues (Gln367 to Lys505), which are linked by 20 residue Pro-rich linker (Gly347 to Thr366). *CbE1*Xyn43-l is predicted to be an extracellular enzyme, with a 26-residue signal peptide (predicted with a probability of 0.8944) preceding the 479-residue deduced amino acid sequence of the mature two-domain full-length enzyme (Fig. 1).

**Fig 2:**
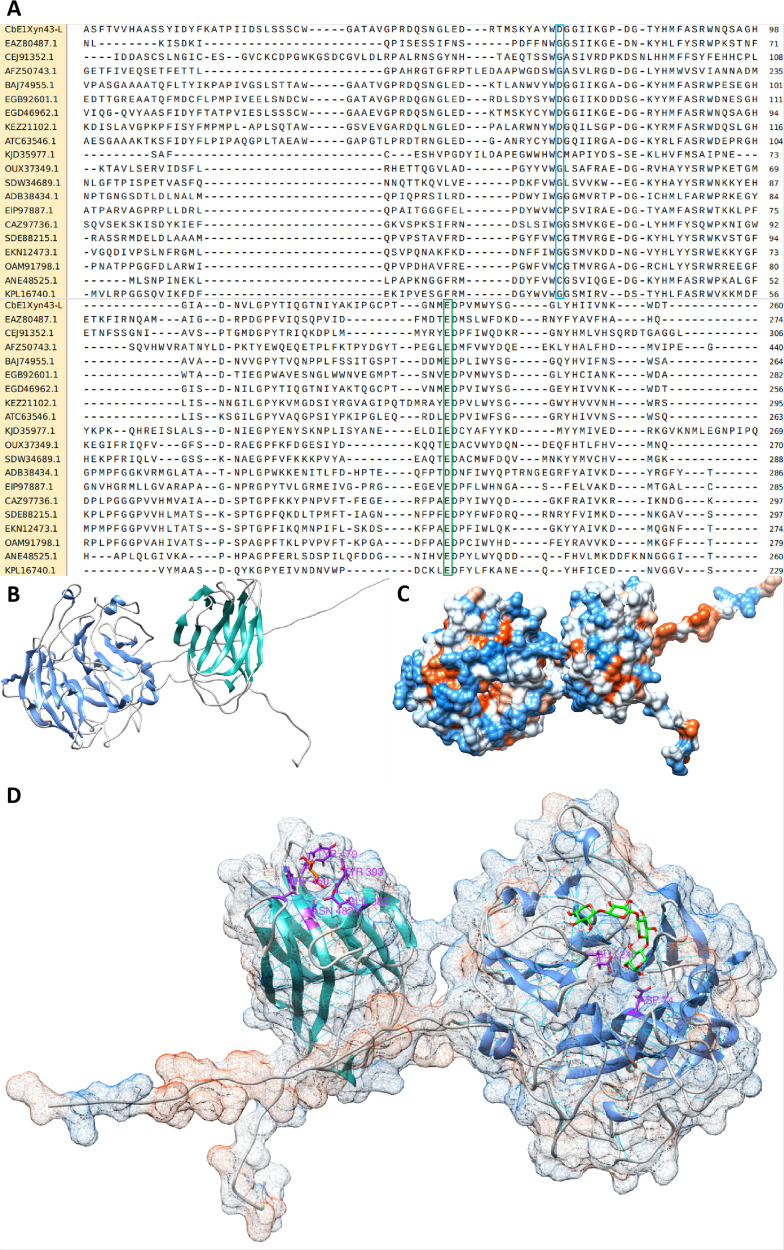
Computational model of *CbE1*Xyn43-l. (**A**) Sequence comparison of selected regions of the catalytic domain of *CbE1*Xyn43-l (E1-CatDom *CbE1*Xyn43-l) with GH43-like enzymes. Two sections are reported: from aminoacids 51–131 (up) and from aminoacids 201–281 (down). The conserved aminoacid residues: base (Asp74) (sky-blue box) and the general acid (Glu240) (green box) are shown according to the catalytic dyad described for GH43-l family. (**B**) Overall structure. It is observed as a two-domain protein. The catalytic domain is represented in sky-blue chain ribbons, and the CBM6 is represented in orange chain ribbons. Overall, the structure shares 72.41% of homology with the template (Model ID AF-A0A4U7JFT2-F1) and 100% of coverage. (**C**) Hydrophobicity surface. It is shown in red the hydrophobic surface and in blue the hydrophilic surface of the protein. (**D**) Details of the interactions between the xylose (shown in green sticks) and CBM6 (shown in orange ribbons) and interactions between xylotetraose (shown in green sticks) and catalytic residues (shown in black sticks) from the catalytic domain (sky-blue ribbons).

The alignment with other GH43-l predicted enzymes allowed the identification of possible catalytic residues belonging to GH43-l family enzymes (Fig. 2A): D74, E240, as well as the conserved residue N190 which may be of importance for substrate accommodation. On the other hand, BLAST on Uniprot and NCBI resulted in the retrieval of 12 hypothetical proteins (with no characterized candidates) covering 90%–100% of the query sequence and displayed sequence identities in the range of 70%–96% to the deduced amino acid sequence of *CbE1*Xyn43-l. The two matches with the highest level of sequence identity were two hypothetical enzymes belonging to *Lacrimispora algidixylanolyticum* (Accession: RKD31825.1) and *Clostridium indicum* (Accession: WP_117419898.1). None of the identified matches corresponded to characterized enzymes, or sequences classified in CAZy database (www.cazy.org), except for the C-terminal CBM6.

For modeling the 3D-structure of *CbE1*Xyn43-l, a cellulase template (Model ID AF-A0A4U7JFT2-F1, AlphaFoldDB) from *Ruminiclostridium herbifermentans* was used, which shares 72.41% of sequence identity and has a coverage of 100% supporting the quality of the model. Furthermore, the structure assessment shows a GMQE of 0.89, with a *Z*-score: −8.61 compared to a *Z*-score: −9.9 of the templates. In addition, ERRAT Complete analysis gave an overall quality factor of 93.3619 and Ramachandran plot shows that 88.7% of the residues are found in the most favored regions, 10.6% are in additional allowed regions, 0.2% in generously allowed regions, and just 0.5% of the residues are found in disallowed regions.

However, because the template used in this modeling does not correspond to a crystallographic model, a final model of the *CbE1*Xyn43-l protein must be constructed using crystallographic data which is beyond the scope of the present study.

The 3D-model of the CBM6 present in *CbE1*Xyn43-l confirmed an overall fold of the jelly roll type, consisting of five antiparallel β-strands on one face and four antiparallel β-strands on the other face connected with loops that vary in length (Fig. 2B). In addition, there are two antiparallel strands close to the loop region. In the loop region, interactions with xylose were predicted involving Tyr393 and Trp450 orientated in parallel to the xylose ring, making a “sandwich” type interaction with the sugar ring, in line with the interactions proposed by Abbott et al. (16) for *Cc*CBM6. Tyr479 (also situated in the loop proposed to be important for carbohydrate recognition, Fig. 2D) makes hydrophobic interaction with xylose, while Glu382 (conserved in many CBM6) and Asn482 make hydrogen bond interactions with the hydroxyl groups connected to C2 and C3 of the xylose (Fig. 2C).

The catalytic domain model reveals a five-bladed β-propeller fold. A comparison of the *CbE1*Xyn43-l amino acids sequence with enzymes of known 3D-structure from 38 other subfamilies belonging to GH43 showed that the position of the active site general base (Asp74) and general acid (Glu240) was conserved, while some literature suggested a third residue in the active site of GH43 enzymes (an Asp, proposed to modulate the pKa of the general acid) (8) was not unambiguously identified here and was replaced by Asn at the corresponding position (Asn190, Fig. 2). However, when *CbE1*Xyn43-l was compared to other 38 hypothetical GH43-l proteins through sequence alignments, all showed this substitution in a conservative manner. Nonetheless, they also show an Asp residue (Asp241 in *CbE1*Xyn43-l), located one position downstream of Glu240 in the amino acid sequence (Fig. 2A) that is fully conserved in most of the hypothetical GH43-like aligned proteins and could have a catalytic function. Proof of this would, however, require a structure of an enzyme from this group of GH43-like enzymes.

### Recombinant production and purification of *CbE1*Xyn43-l

To produce *CbE1*Xyn43-l in active form, the predicted signal peptide was removed and replaced by a Met-residue, a His-tag with the sequence LeuGluHisHisHisHisHisHis (Fig. 1) was added to the C-terminal of the protein gene sequence. Finally, *CbE1*Xyn43-l gene was cloned in the vector pET21a and the corresponding enzyme was produced in *E. coli* Rosetta-Gami (DE3).

**Fig 3:**
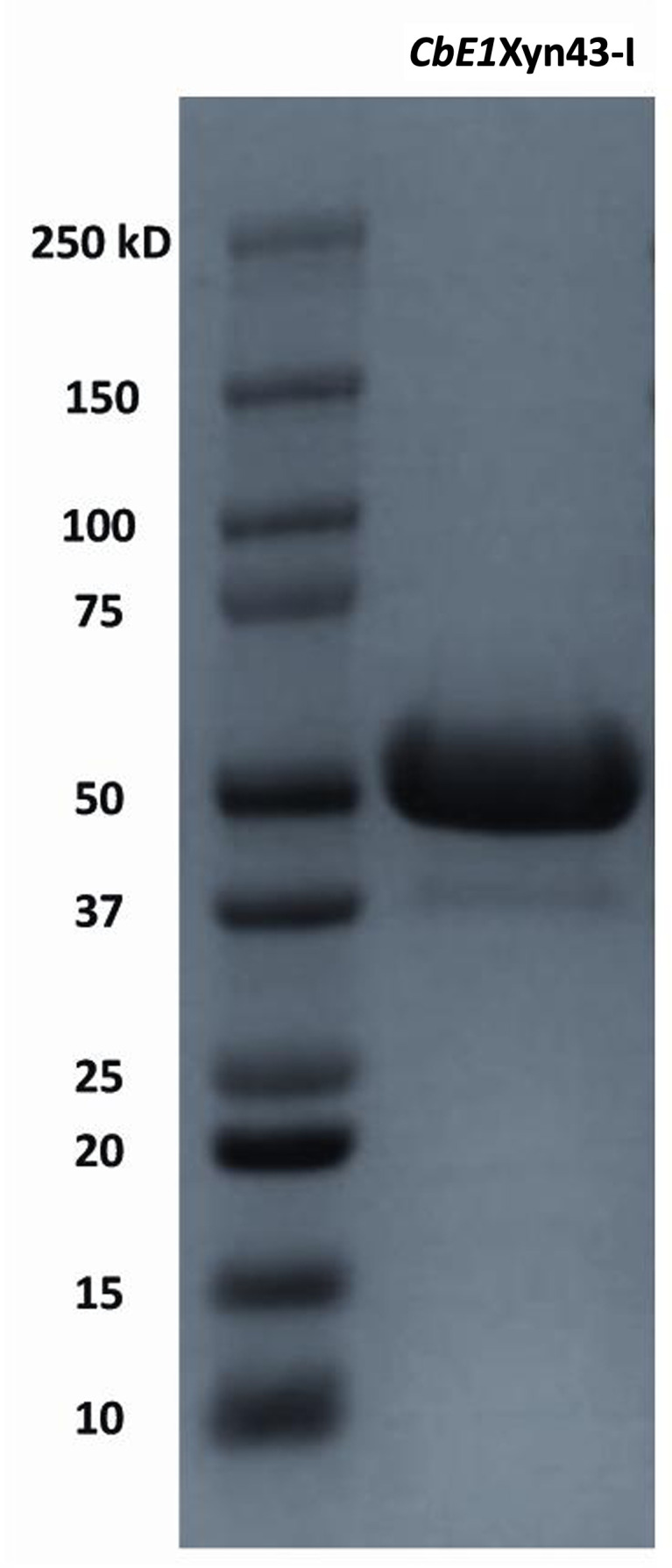
SDS PAGE of the studied *CbE1*Xyn43-l from *C. boliviensis* strain E-1, MW 52.9 kDa.

The molecular weight of the target protein (including the C-terminal His tag) was estimated to 52.9 kDa (Fig. 3), in line with the theoretically determined molecular weight of 52.8 kDa (based on the amino acid sequence and including the C-terminal His-tag). After purification by immobilized metal ion affinity chromatography and dialysis, a 2.0 mg mL^−1^ protein of approximately 90% purity was obtained and stored at 4°C.

### Biochemical characterization of *CbE1*Xyn43-l using aryl substrates

The activity of *CbE1*Xyn43-l was screened using a number of synthetic substrates. The activity was only instead detected using the substrate *p-*nitrophenyl xylobioside (*p*NPX_2_) confirming endo-xylanase activity of *CbE1*Xyn43-l (Table 1). This substrate was subsequently used to determine its temperature and pH optima and to retrieve data on enzyme kinetics and residual activity.

**TABLE 1:** Specific activity of *CbE1*Xyn43-l measured on aryl and natural substrates (mean ± SD) (*n* = 3) using *p*NPX_2_ and the 3,5-dinitrosalysilic acid (DNS) assays for activity detection

Substrate	Specific activity (*n* = 3) (U mg^−1^)*^a^*
*p*-Nitrophenyl xylopyranoside (*p*NPX)	ND^*b*^
*p*-Nitrophenyl arabinofuranoside (*p*NPA)	ND
*p*-Nitrophenyl glucopyranoside (*p*NPG)	ND
*p*-Nitrophenyl galactopyranoside (*p*NPGal)	ND
*p*-Nitrophenyl mannopyranoside (*p*NPM)	ND
*p*-Nitrophenyl xylobioside (*p*NPX_2_)	0.16 ± 0.02
Quinoa bran arabinoglucan	ND
Debranched arabinan	ND
Cellulose	ND
Starch	ND
Xyloglucan	ND
Birchwood xylan	0.40 ± 0.01
Quinoa stalks glucuronoarabinoxylan	0.29 ± 0.01
Wheat arabinoxylan	0.51 ± 0.03

^*a*^ One unit is defined as the amount of enzymes required for producing 1 µmol of xylose-equivalents per minute under optimized temperature and pH parameters (65°C, pH 7.0).

^*b*^ ND, not determined.

**Fig 4:**
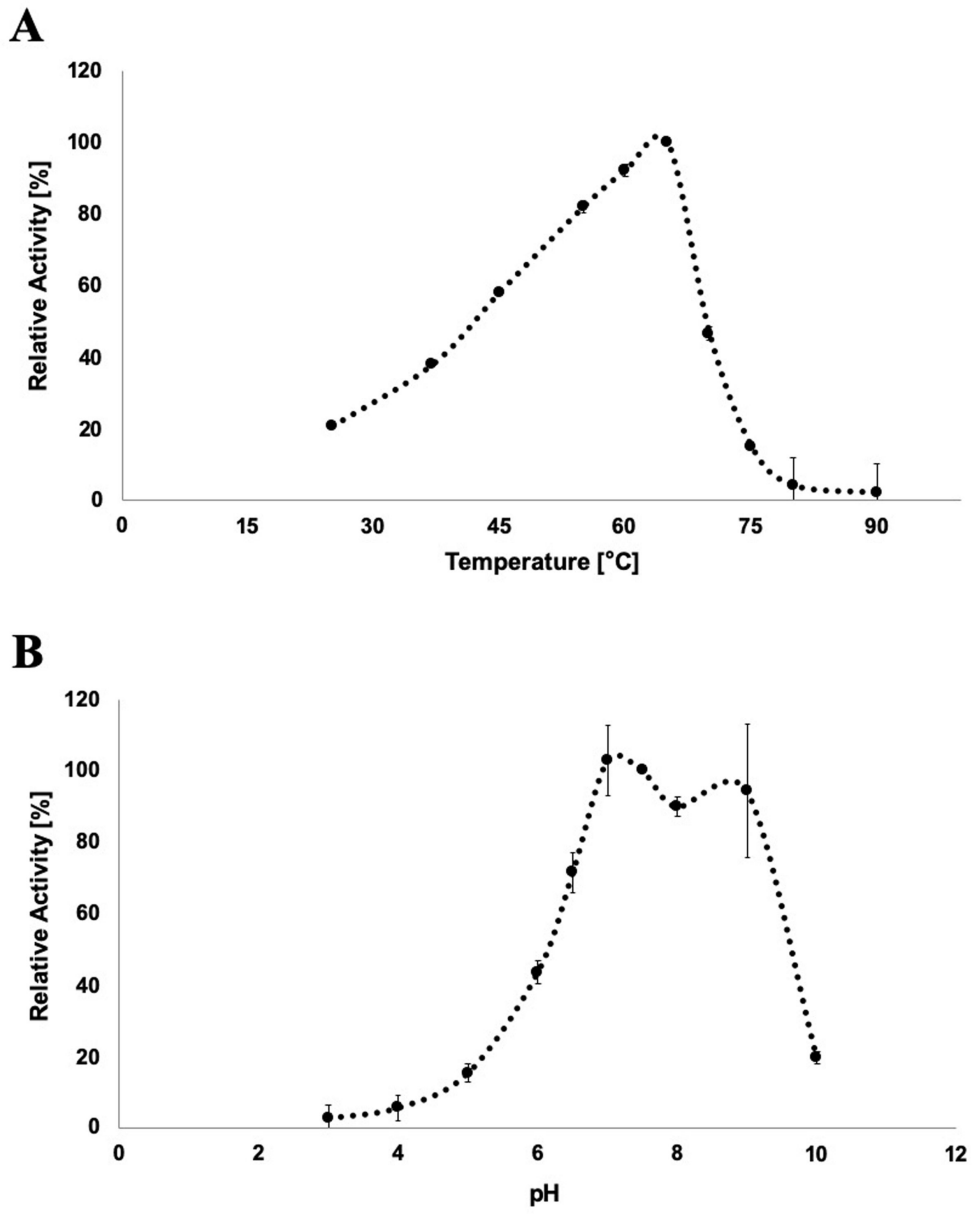
(**A**) Optimum temperature and, (**B**) optimum pH determined for *CbE1*Xyn43-l through a thermocycler-microtube assay using *p*NPX_2_ as substrate. The highest records observed at 65°C (**A**) and at pH 7 (**B**) were set as 100%.

*CbE1*Xyn43-l showed maximum product release at 65°C in the presence of the substrate, and activity was considerably decreased at both lower and higher temperatures (e.g., 39% of activity was detected when the temperature was decreased to 37°C, and 55% of the activity was detected when the temperature was increased to 70°C, in both cases using the same incubation period). Activity was almost totally lost at 80°C (Fig. 4A).

A broad pH optimum was observed, but the activity was affected by the buffer used. In McIlvaine buffer, optimum pH was established at 7.0, but use of Glycine/NaOH buffer at pH 9.0 resulted in higher activity than that observed at pH 8.0 in the McIlvaine buffer system (Fig. 4B).

Michaelis-Menten kinetic parameters were estimated through nonlinear regression using substrate concentration and reaction velocity data giving an *R*^2^ value of 0.93. The *K*_m_ value could not be determined accurately because it was below the lowest substrate concentration used [0.5 mM]. On the other hand, the *k*_cat_ value of *CbE1*Xyn43-l was determined to be 0.044 s^−1^ for *p*NPX_2_ substrate.

For measurements of residual activity, *CbE1*Xyn43-l was constantly incubated for 168 h (7 days) at 65°C to measure thermal stability, using irreversible deactivation analysis, at its observed optimum temperature. After the incubation period, the samples were removed and subjected to the standard activity analysis. *CbE1*Xyn43-l activity was maintained constant, or even increased, after the complete incubation time. In fact, *CbE1*Xyn43-l showed an increase of activity after 7 days to 113%.

In addition, *CbE1*Xyn43-l also showed good storage stability at 4°C and could be stored for >6 months without loss of activity (data not shown).

### Specific activity of *CbE1*Xyn43-l using arabino/glucuronoxylans substrates

From the eight natural substrates measured to determine the activity of *CbE1*Xyn43-l, only the three different types of xylans (birchwood xylan, quinoa stalks glucuronoarabinoxylan, and wheat arabinoxylan) showed catalytic activities (Table 1). *CbE1*Xyn43-l showed higher specific activity on the three xylan-types than on *p*NPX_2_, and although the specific activity was low (not exceeding 1 U mg^−1^) (Table 1), the verified activity on the xylan polymers, motivated analysis of the hydrolysis product pattern, to investigate the possibility to utilize this enzyme for production of XOs.

**Fig 5:**
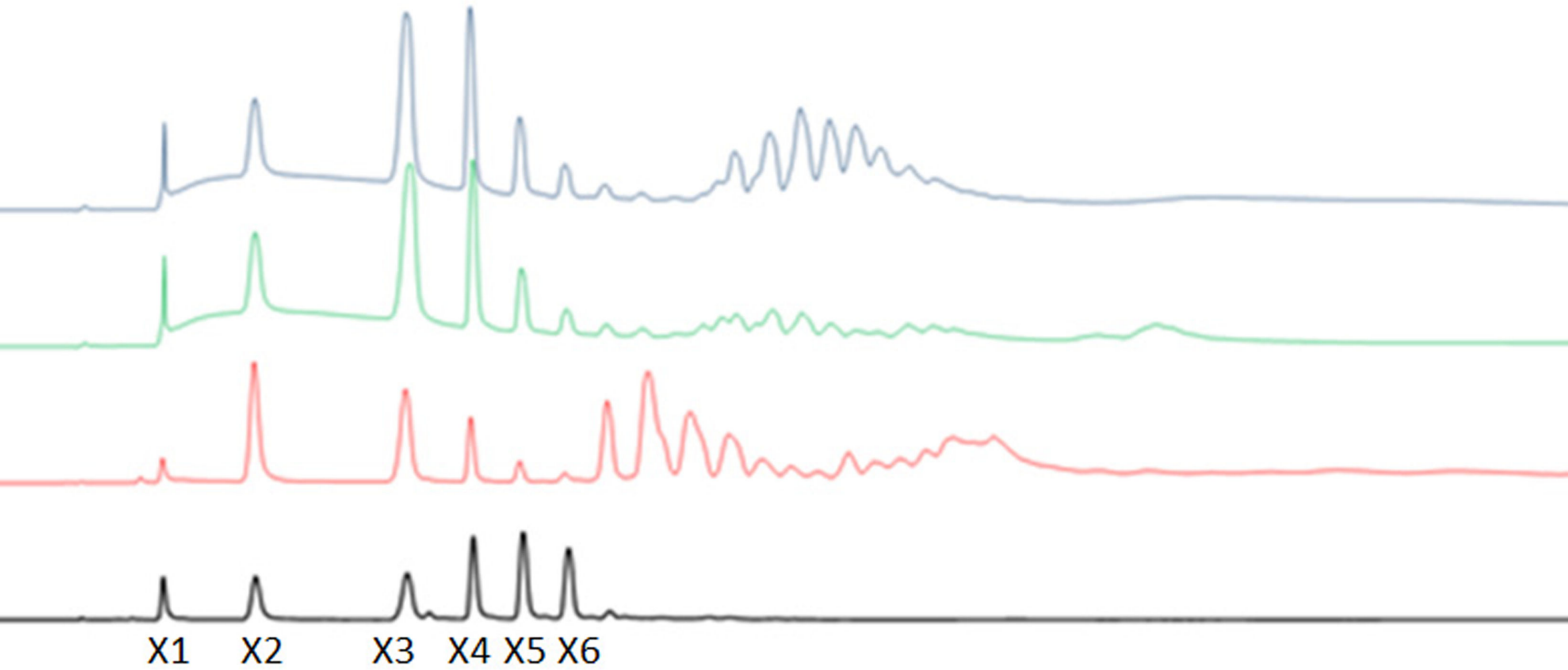
Xyligosaccharide (XO) peaks detected by HPAEC-PAD. The product profile of *CbE1*Xyn43-l after hydrolysis of birchwood xylan (green), quinoa stalks glucuronoarabinoxylan (blue), and debranched wheat arabinoxylan (red) are shown. XO standards are from Megazyme (black), and the peaks from left to right show: xylose (**X1**), xylobiose (**X2**), xylotriose (**X3**), xylotetraose (**X4**), xylopentaose (**X5**), and xylohexaose (**X6**).

Production of XOs was measured by HPAEC-PAD, after separate hydrolysis trials of *CbE1*Xyn43-l on the three different xylan (birchwood xylan, quinoa stalks glucuronoarabinoxylan, and wheat arabinoxylan). The most common oligosaccharide products after hydrolysis by *CbE1*Xyn43-l were xylobiose, xylotriose, and xylotetraose. *CbE1*Xyn43-l produced minimal amounts of xylose on birchwood xylan and quinoa stalks glucuronoarabinoxylan (Table 2). Detectable amounts of xylose were produced when wheat arabinoxylan was used. The product profile also showed a number of unidentified oligosaccharides from the substrates (Fig. 5).

**TABLE 2:** Xylooligosaccharide profile produced by *CbE1*Xyn43-l using Birchwood xylan, Quinoa stalks glucuronoarabinoxylan, and Wheat arabinoxylan as substrates

Substrate	Yield (mg of XOs/g of substrate) (*n* = 3) (relative activity [%]*^a^*)					
	X1	X2	X3	X4	X5	X6
Birchwood xylan	0.02 ± 0.00 (0)	0.42 ± 0.01 (21.0)	0.51 ± 0.03 (25.7)	0.46 ± 0.02 (23.1)	0.30 ± 0.01 (14.9)	0.31 ± 0.01 (15.3)
Quinoa stalks glucuronoarabinoxylan	0.01 ± 0.01 (0)	0.40 ± 0.01 (21.5)	0.55 ± 0.10 (30.1)	0.45 ± 0.02 (24.6)	0.22 ± 0.02 (12.0)	0.22 ± 0.01 (11.8)
Wheat arabinoxylan	0.07 ± 0.01 (0)	0.12 ± 0.01 (23.9)	0.17 ± 0.01 (34.4)	0.12 ± 0.01 (23.0)	0.05 ± 0.01 (10.0)	0.05 ± 0.01 (8.8)

^*a*^ Relative amount [%] was determined dividing the mean value of each XOs (xylose was excluded as not being considered an oligosaccharide) with the sum of the determined XOs.

To evaluate the effect of arabinosylation on the activity of *CbE1*Xyn43-l, 3^3^-α-l-arabinofuranosyl-xylotetraose (XA^3^XX), 2^3^-α-l-arabinofuranosyl-xylotriose (A^2^XX), and 2^3^,3^3^-di-α-l-arabinofuranosyl-xylotriose (A^2,3^XX) were used as substrate. From these substrates, *CbE1*Xyn43-l was only able to hydrolyze the arabinosylated xylotetraose XA^3^XX and released the reducing end xylopyranose but was unable to hydrolyze the arabinofuranosyl α- (1–3) substituent in the substrate, showing that *CbE1*Xyn43-l possessed xylanase activity, but no arabinofuranosidase activity. A^2,3^XX and A^2^XX were not hydrolyzed by *CbE1*Xyn43-l (data not shown), indicating that the enzyme may need three unsubstituted xylose residues in a row for activity. The enzyme did not display activity on α- (1–5) linked arabinan either (Table 1).

### Specific activity of *CbE1*Xyn43-l on arabino/glucuronoxylans in the presence of divalent ions

Divalent cations (1 and 10 mM) were evaluated to test potential interaction with the enzyme to improve the specific activity of *CbE1*Xyn43-l on the xylan substrates (birchwood xylan, quinoa stalks glucuronoarabinoxylan, and wheat arabinoxylan) and also to determine any possible inhibitory effect in its catalytic capacity in the presence of EDTA.

The addition of divalent ions did have some effect on specific activity of *CbE1*Xyn43-l but was dependent on the type and concentration of the ion, as well as the type of xylan used in the assay. Mg^2+^ and Fe^2+^ increased the activity of *CbE1*Xyn43-l on the xylan substrates, but with least effect on wheat arabinoxylan. The addition of Cu^2+^ resulted in a significant reduction of the activity on all substrates used. Presence of EDTA generally led to a small reduction of the specific activity, but this effect was less significant than the decrease observed upon the addition of Cu^2+^ (Table 3).

**TABLE 3:** Effect of divalent metal ions (and EDTA) on the hydrolysis of different xylans by *CbE1*Xyn43-l, using the 3,5-dinitrosalicylic acid assay as method of detection. Activity is shown as relative activity (%), compared to a control without added ion/EDTA

Metal ion	Relative activity (*n* = 3) (%)					
	Birchwood xylan		Quinoa stalks glucuronoarabinoxylan		Wheat arabinoxylan	
	(1 mM)	(10 mM)	(1 mM)	(10 mM)	(1 mM)	(10 mM)
Mg^2+^	121.7 ± 5.7	107.8 ± 2.7	105.5 ± 4.1	106.9 ± 9.2	104.7 ± 7.3	101.4 ± 6.3
Cu^2+^	35.3 ± 0.3	20.5 ± 1.7	65.9 ± 5.1	24.8 ± 0.8	38.7 ± 2.7	20.4 ± 0.8
Zn^2+^	98.4 ± 11.7	77.1 ± 11.8	105.0 ± 1.8	94.3 ± 3.6	94.9 ± 3.7	69.8 ± 3.8
Ca^2+^	90.8 ± 4.9	89.8 ± 0.8	103.8 ± 3.1	109.1 ± 1.4	93.4 ± 3.3	100.3 ± 10.9
Fe^2+^	112.8 ± 5.4	111.0 ± 10.7	134.8 ± 14.9	144.2 ± 2.3	102.4 ± 3.4	87.4 ± 18.1
EDTA	88.1 ± 3.8	76.3 ± 0.8	105.3 ± 2.7	96.4 ± 1.9	85.1 ± 4.7	64.2 ± 5.4
Control	100 ± 0	100 ± 0	100 ± 0	100 ± 0	100 ± 0	100 ± 0

Certain differences were also observed between the three polymeric xylan substrates. Assays using quinoa stalks glucuronoarabinoxylan as substrate were generally less affected at the low concentration of the respective ion, except for Fe^2+^ where an activity increase was significant already at 1 mM. The activity of quinoa xylan was also slightly increased by the higher addition (10 mM) of Ca^2+^. This may indicate that differing amounts of ions were present in the substrates from the beginning or that the different substituents in the xylans led to different types of interactions. Further metal ion analysis of the substrates is necessary to verify this pattern.

## DISCUSSION

Glycoside hydrolases (GHs) (EC 3.2.1.-) are extremely common enzymes, with a number of roles in nature, that include degradation of the major polymers in biomass (such as cellulose, hemicellulose, and starch) in anti-bacterial defense strategies, in pathogenesis mechanisms as well as in normal cellular function (e.g., trimming of glycoprotein). The Carbohydrate Active Enzymes Database (www.cazy.org) provides a continuously updated list of the glycoside hydrolase families, based on amino acid sequence and folding similarities. Despite the enormous effort in classifying these enzymes into different families, there are, with the continuously increasing genetic material from various sequencing projects, still groups of enzymes that are not yet classified into these families.

In this work, we have cloned, produced, and characterized an enzyme annotated as GH43-like. It belongs to a large subgroup of hypothetical protein found in different sequence databases but is not classified in CAZy. The sequences of these hypothetical proteins share weak sequence similarity with the GH43 family. The GH43-like enzyme, characterized here (*CbE1*Xyn43-l), shows limited sequence similarity (less than 20% id) to some of the subfamilies in GH43 (subfamily 4, 5, and 12). In addition, the two residues in the catalytic dyad of GH43 are conserved, while the literature suggested third residue (believed to play a role in pKa modulation of the general acid and to correct the orientation of the substrate and general acid) (10) was found to be an Asn190. However, as the sequence of *CbE1*Xyn43-l is closely related to a large number of hypothetical proteins annotated as GH43-like (38 sequences of GH43-l were used in the present study for the comparison), we have investigated the function of this enzyme and found that it displays endo-xylanase activity.

The genome of *C. boliviensis* strain E-1 did not contain genes encoding enzymes from the well-known xylan degrading enzymes in GH10 and GH11. Moreover, no candidate genes encoding GH5 arabinoxylanases or GH30 glucuronoxylanases could be identified. Hence, it was hypothesized that the GH43-like enzyme described here could be the enzyme that allowed the microorganism to grow on xylan (15). The presence of a clearly defined signal peptide according to Signal P predictions supports the export of the enzyme, which may function as a first degradation step on the xylan polymer that allows uptake of produced oligosaccharides in the microorganism. The enzyme has homologs in many related *Clostridium* strains which may indicate a similar function and be previously unknown type of endo-xylanase in this evolutionary lineage of microorganisms.

The use of different types of substrates showed that the most common hydrolysis product has a degree of polymerization of 3 (or 4), but that the enzyme can degrade DP3 further, although with low catalytic efficiency. Oligosaccharides with DP3 and 4 have previously been identified from cultivation media of *C. boliviensis* strain E-1 when grown on commercial xylan as sole carbon source (Salas-Veizaga, unpublished data), indicating a corresponding profile to the hydrolysis profile of the enzyme characterized here.

This enzyme, despite not catalyzing substituent decoration(s) on the substrate, seems to produce substituted and unsubstituted oligosaccharides as an additional activity, suggesting that *CbE1*Xyn43-l released different oligosaccharide products as its endo-xylanase activity, which is evident from the product profiles shown in the HPAEC-PAD analysis, where a number of unidentified oligosaccharides were visible. The pattern of unidentified peaks differed between the xylans substituted with only arabinose (wheat) or with glucuronic acid and arabinose (quinoa), showing that both types of substituents must not interfere in the active site. Linkages that are common for arabinofuranoside residues in terrestrial plants are α- (1–5) in arabinans, and α- (1, 2) and α- (1–3) in arabinofuranosyl residues linked to the xylopyranose backbone of xylans (i.e., arabinoxylans) (7, 11). No arabinose was, however, released from either α- (1–5), α- (1, 2), or α- (1–3) linked residues. Moreover, the use of α- (1–3) substituted AXOs with a xylotetraose backbone only led to the release of xylose. This shows that some arabinose substituents can be accepted in the binding site of the enzyme but that three unsubstituted xylose residues seemed important for hydrolysis activity.

Subfamilies 12, 16, and 29 from GH43 family have enzymes described with endo-xylanase activity (CAZy database 2023-10-16). Some of the enzymes displaying endo-xylanase activity are, however, multifunctional, also acting as xylosiadases or arabinofuranosidases (17, 18). A putative enzyme (genbank ACM91046.1, classified under subfamily 29) from the uncultured bacterium URE4 is to date also annotated as endo-xylanase, similar to *CbE1*Xyn43-l, and described as a two-domain protein composed by a CBM family 6 and a GH43 catalytic domain (in UniProtKB-COK016). No characterization data is, however, yet available, making the annotation uncertain.

*CbE1*Xyn43-l showed a maximum activity of 0.4 U mg^−1^ using birchwood xylan. This is low in comparison with the activity of the thermostable GH10 enzyme *Rm*Xyn10A that had an activity of approximately 120 U mg^−1^ on the same substrate (19, 20). However, the activity level varies significantly between enzymes and their families, and for example, the GH43 xylanase from *Paenibacillus curdlanolyticus* strain B-6 also displayed rather low specific activity using the same substrate (2.9 U mg^−1^ on birchwood) (18).

The temperature optimum and long stability in irreversible deactivation studies contrast the optimum growth temperature of the organism. *C. boliviensis* strain E-1 is a mesophilic anaerobe, shown to have a growth optimum at 37°C (15) and isolated from a riverbank at even lower temperature. Optimum pH, on the other hand, correlates with the optimum growth pH of the strain (pH 6.8–7.4) (15). As this enzyme has a signal peptide, it is most likely extracellular, and extracellular enzymes are commonly reported to be more resistant to increased temperatures than their native host organisms.

Compared to the product profile of xylan degrading enzymes from other GH families, *CbE1*Xyn43-l produced oligosaccharides with higher DP than the most common hydrolysis products from GH10 and GH11 (DP2 and DP3) (21–23). The X2, X3, and X4 production combined with almost null xylose production makes *CbE1*Xyn43-l a candidate for prebiotic XO production. Allowing limited downstream processing prior to use as food-grade XOs, considering the costs of separation of monomeric and oligomeric products (24). Moreover, substituted-XOs are of interest as they may introduce selectivity among probiotic bacteria.

### Conclusions

*CbE1*Xyn43-l has here been described as a strict endo-xylanase with the capacity to produce xylotriose and xylotetraose as main products using different xylan types. The sequence analysis and the modeling approach here estimated showed both catalytic and CBM6 domains of the protein, similarity search and alignments show that the enzyme is a member of a large group of hypothetical proteins that are annotated as GH43-like. Moreover, the general base (Asp74) and general acid (Glu240) in the catalytic dyad characteristic of GH43 were conserved in the catalytic domain. Divalent ions as well as EDTA affected the activity of *CbE1*Xyn43-l, indicating a metal ion binding site. The production of unidentified oligosaccharides (along with linear XOs) suggests that substituted-XOs are being produced by *CbE1*Xyn43-l, not only indicating acceptance of substituents in the active site of the enzyme but also requiring unsubstituted xylose residues. This pattern of peaks opens the possibility to produce various sets of oligosaccharide products that could be of importance for prebiotics production.

## MATERIALS AND METHODS

### Bacterial strains

*Clostridium boliviensis* strain E-1 (=CCUG 50824^T^, =DMS 17227^T^) (AY943862) (NCBI: JAWONS000000000.1)*,* referred to in the rest of the present research article as E-1 strain, was isolated and first described by Álvarez Aliaga (15).

The strain for cloning was *E. coli* NovaBlue (Novagen, Birmingham, UK), and the strain for recombinant production was *E. coli* Rosetta-Gami (DE3) (Novagen).

### Genome sequencing of *C. boliviensis* strain E-1

The strain was grown in a modified PYG medium (DMSZ, German Collection of Microorganisms and Cell cultures GmbH, Germany). The medium consisted in (g L^−1^): Trypticase peptone (5), peptone (5), yeast extract (10), meat extract (5), glucose (5), K_2_HPO_4_ (2), Tween 80 (1 mL), cystein-HCl.H_2_0 (0.5), resarzurin (1 mg), salt solution (40 mL), and vitamin solution (0.2 mL). Salt solution (g L^−1^): CaCl_2_·2H_2_O (0.25), MgSO_4_·7H_2_O (0.5), K_2_HPO_4_ (1), KH_2_PO_4_ (1), NaHCO_3_ (10), NaCl (2). Vitamin solution (mg L^−1^): biotin (10), *p*-aminobenzoic acid (50), vitamin B-12 (50), thiamine (100). Overnight anaerobic cultures were concentrated, and then, the cell pellet was used to extract the genomic DNA using ZR Fungal/Bacterial DNA Mini-Prep-Metagenomics Company (Zymoresearch, USA) to purify a minimum of 3 µg of DNA.

A library for the novo genome sequencing was made using the TruSeq PCR-Free method (Illumina San Diego, CA, USA) according to TruSeq PCR-Free Sample Preparation Guide (#15036187 Rev. B) with the exception that genomic DNA was fragmented in a fragmentase reaction. A second Library was made using the TruSeq Nano method according to the associated Reference Guide (#15041110 Rev. D) with the exception that genomic DNA was fragmented using nebulization. The two libraries were sequenced on separate runs on the Illumina MiSeq sequencing platform, using V3 600 cycle sequencing chemistry (Illumina, USA).

Overlapping reads for each library were merged using FLASH (v.1.2.11) (25), and both merged and unmerged reads for both libraries were used in assembly. *De novo* assembly of the draft genome was done using the GS *De Novo* Assembler (v2.9, Roche), and the resulting draft genome was annotated using the online RAST annotation server (26) and used for gene retrieval.

To improve the genome assembly for publication, a third sequencing library was made using the Nextera Mate Pair method according to the associated Reference Guide (#15035209 v02) following the gel-free protocol and with the exception that DNA fragments were cleaned up using the NucleoSpin gDNA Cleanup columns (Macherey-Nagel). This library was sequenced on the MiSeq sequencing platform, using the V2 500 cycle sequencing chemistry (Illumina). All reads from this Mate Pair library and both previous shotgun libraries were trimmed to quality using Trimmomatic (v.0.36) (27), the trimmed reads assembled using SPAdes (v.3.12.0) (28), and this improved draft genome annotated by the RAST server.

### Bioinformatics and sequence alignment

The predicted genes were functionally annotated first by alignment using DIAMOND (v2.0.15.153) (29) against Uniprot database (https://www.uniprot.org/) (update 2023–09-30) with a ID% = 80% and e = 10^−5^ and were submitted to GhostKOALA (v2.0) (30) and eggNOG-mapper (v2.1.12) (31) for orthology assignment. Furthermore, all the predicted genes were analyzed using InterProScan (v5.64-96.0) (32) locally in order to access the classification of protein families. The GH43 putative identified genes were retrieved manually and forward submitted to dbCAN3 server (v3.0.1) (33) for Glycoside hydrolase family confirmation.

The presence of a signal peptide was predicted using the SignalP (v6.0) (34) with a setup as follows: gram positive and slow-sequential mode.

All the identified candidates were then submitted to BLASTp at NCBI (https://blast.ncbi.nlm.nih.gov/Blast.cgi?PAGE=Proteins) and Uniprot BLAST (https://www.uniprot.org/blast) to choose the most novel candidate.

For alignments, one structure determined member each, from 39 different subfamilies of GH43 in the CAZy database (http://www.cazy.org/), was selected. Amino acid sequences of these proteins were retrieved from the Uniprot and NCBI databases (Uniprot and NCBI, update 2023-10-16) and aligned with the catalytic domain of *CbE1*Xyn43-l using the program Clustal Omega (v1.2.4) (35) to identify sequence similarities and conserved residues. Furthermore, the amino acid sequence of *CbE1*Xyn43-l was submitted to CD-Search at NCBI (36) in order to access to GH43 subclassification and identify the corresponding dyad of catalytic residues. Next, 19 members of identified subclassification were retrieved from NCBI and together *CbE1*Xyn43-l were aligned using the program Clustal Omega (v1.2.4) (35) and visualized using SnapGene Viewer . Additionally, *CbE1*Xyn43-l was aligned retrieved aminoacids sequences by DIAMOND (v2.0.15.153) (29) for identifying shared homology.

Prediction of molecular weight and molar absorption coefficient was made using the ProtParam tool at https://web.expasy.org/protparam/. The molar absorption coefficient was predicted to be 112 105 M^−1^ cm^−1^.

### Molecular modeling

The three-dimensional structure of the enzyme was obtained by homology modeling using the web server SWISS-MODEL (37). The accuracy of the modeled structure was supported by the relatively high identity in amino acid sequences of the template and was further evaluated by *Z*-score through ProSA-web server (38), stereochemical parameters of the protein structure and Ramachandran plots were predicted using ERRAT (39) and PROCHECK (40) on SAVES (v6.0) online server (https://saves.mbi.ucla.edu/), and protein structure was further assessment by Structure Assessment online tool (37).

Molecular docking was performed using Autodock Vina (v1.1.2) (41) on UCSF Chimera (v1.17.3) (42) taking as ligand d-Xylose for CBM6 docking and d-Xylotriose for catalytic domain.

### Cloning and recombinant production of *CbE1*Xyn43-l

The gene encoding *CbE1*Xyn43-l was PCR amplified using parameters derived from the genome sequence data (Table 4) for cloning in frame with the C-terminal His tag in the expression of plasmid pET21a. The gene was initially cloned in the propagation vector pUC19 giving the plasmids pUC19::Xyn43-l and then subcloned in the expression vector pET21a, between the restriction sites Ndel (including a Met start codon) and Xhol, resulting in the plasmid pET21a::Xyn43-l.

**TABLE 4:** Primers used for cloning the gen codifying *CbE1*Xyn43-l

Gen	Sequence 5′ to 3′	Length
*CbE1*Xyn43-l F	gctagcTCATCGTATATTGATTATTTTAAAGCGACGC	37
*CbE1*Xyn43-l R	ctcgagTTTGCCCATGGAATCTAAATCACCC	31

The expression vector was first introduced into chemical competent *E. coli* BL21 (DE3), Rosetta and, finally, Rosetta-Gami (DE3) strains (Novagen) by thermic shock. Only Rosetta-Gami (DE3) resulted in an active recombinant enzyme production; then, the recombinant strain was grown in LB medium supplemented with ampicillin sodium salt (100 µg mL^−1^) and chloramphenicol (20 µg mL^−1^). The production of the protein was induced with 1 mM IPTG at room temperature and cultivated overnight with constant shaking at 200 rpm. The cell pellet was resuspended in sodium phosphate buffer (20 mM)/NaCl (500 mM) buffer (pH 7.4) and sonicated to disrupt the cell walls. The produced enzyme was purified by affinity chromatography (IMAC) using a GE Health Care Life Sciences ÄKTA system (Uppsala, Sweden) and a His Trap FF Sepharose 6 Column (GE-Healthcare). The column was equilibrated and washed with binding buffer (100 mM phosphate, 500 mM NaCl, pH 7.4), and the target protein was eluted with an imidazole containing elution buffer (100 mM phosphate, 500 mM NaCl, 500 mM imidazole, pH 7.4). Finally, the enzyme was dialyzed in binding buffer without imidazole to eliminate the excess of imidazole.

### Substrate screening using aryl substrates, pH, and temperature optima of *CbE1*Xyn43-l

Xylanase activity was measured using *p-*nitrophenyl xylobioside (*p*NPX_2_) (Megazyme, Bray, Ireland). To measure possible xylosidase or arabinofuranosidase or any further activities, *p-*nitrophenyl xylopyranose (*p*NPX), *p*-nitrophenyl arabinofuranoside (*p*NPA), *p-*nitrophenyl glucopyranoside (*p*NPG), *p-*nitrophenyl galactopyranoside (*p*NPGal), and *p-*nitrophenyl mannopyranoside (*p*NPM) were used (Sigma Aldrich, Stockholm, Sweden). Reaction time was first determined. In a 96-well plate using 1 mM of *p*NPX_2_ in phosphate/Na buffer, 50 mM (pH 7.0) was mixed with *CbE1*Xyn43-l (20 µM). The plate then was incubated at 37°C, and changes in absorbance at 400 nm were measured at 5 min intervals until the absorbance reached a saturation point. After that, half of the time required to reach the saturation point was used as the reaction time to establish temperature and pH optima using the same substrate in the following reactions.

To establish the optimum temperature and pH, micro-reactions using PCR microtubes and Thermocycler T-Gradient (Biometra, Göttingen, Germany) were used. A dilute concentration of the enzyme (20 µM) was added to 1 mM of *p*NPX_2_ substrate contained in phosphate/Na 50 mM (pH 7.0). The tubes were then placed in the thermocycler, establishing a gradient of temperatures that included 25, 37, 45, 55, 60, 65, 70, 75, 80 and 90°C.

Optimum pH was also tested using PCR micro-tubes assay, 1 mM of *p*NPX_2_ in McIlvaine buffers 50 mM at pH 3, 4, 5, 6, 6.5, 7, 7.5, 8 and in glycine/NaOH buffers 50 mM at pH 9 and 10 were mixed with 20 µM of *CbE1*Xyn43-l.

The reaction times were 90 min in assays determining optimum pH and temperature, and triplicate samples and controls without enzyme were assayed. Once the assays were finished, the total volumes of the microtubes were transferred to 96-well plate and the absorbance was measured at 400 nm using Multiskan Go 96-Plate reader (Thermo Scientific, Gothenburg, Sweden).

### Estimation of kinetic parameters using *p*NPX_2_

The kinetic parameters were estimated using *p*NPX_2_ in concentrations of 0, 0.5, 1, 2, 3, 5, and 10 mM. In 96-well plate, the mentioned concentrations of *p*NPX_2_ were prepared in phosphate/Na buffer 50 mM (pH 7.0) and incubated in the presence of *CbE1*Xyn43-l (20 µM) at 65°C. The change in absorbance (400 nm) was measured at 5 min intervals for 240 min.

For each concentration, triplicates and also controls without enzyme were analyzed. Michaelis-Menten kinetic parameters were estimated by nonlinear regression, using the software Renz Package 0.2.1 within R studio v.4.2, with the substrate concentration (*S*) and reaction velocities (*V*_0_) as input values.

### Residual activity (irreversible deactivation) assay

*CbE1*Xyn43-l (20 µM) was incubated for 168 h (7 days) at 65°C at incubation times of 0, 6, 12, 18, 24, 48, 72, 96, and 168 h. After the incubation, 1 mM *p*NPX_2_ in phosphate/Na 50 mM (pH 7.0) was mixed with the enzyme. Subsequently, the reactions were assayed for 90 min at 65°C. Finally, the absorbance was measured at 400 nm. The activity measured at 0 h time was considered 100% of activity.

### Specific activity of *CbE1*Xyn43-l using arabino/glucuronoxylans substrates

Specific activity of *CbE1*Xyn43-l was assayed using the 3,5-dinitrosalysilic acid (DNS) method (43). One percentage (wt/vol) of quinoa bran arabinoglucan, debranched arabinan (Megazyme), cellulose (Sigma-Aldrich), starch, xyloglucan (Megazyme), birchwood xylan (Sigma Aldrich), quinoa stalks glucuronoarabinoxylan (13), and wheat arabinoxylan (Megazyme) was dissolved in 50 mM phosphate/Na buffer (pH 7.0). The substrates were mixed with *CbE1*Xyn43-l (20 µM) and incubated for 90 min at 65°C, and phosphate/Na buffer was used as negative control. Once the reaction was finished, DNS was added and the samples were boiled for 10 min. The samples were then transferred to a 96-well plate and absorbance was measured at 540 nm. Standard curves to determine the specific activity in terms of xylose equivalents were made using 1% (wt/vol) of each substrate mixed with known concentrations of d-Xylose (Sigma Aldrich).

### Activity of *CbE1*Xyn43-l on arabino/glucuronoxylans in the presence of metal ions

*CbE1*Xyn43-l (20 µM) was assayed in the presence of either of the metal ions Mg^2+^, Cu^2+^, Zn^2+^, Ca^2+^, Fe^2+^, and also EDTA. Birchwood xylan, quinoa stalks glucuronoarabinoxylan, and wheat arabinoxylan (1%, wt/vol), respectively, were dissolved in phosphate/Na buffer 50 mM (pH 7.0) and mixed with each of the mentioned metal ions or with EDTA at concentrations of 1 and 10 mM. Hydrolysis reactions were subsequently made at 65°C for 90 min. The DNS method was used to determine the variation in specific activity. Reactions in the absence of metal ion were considered 100% of enzyme activity.

### Effect of arabinosylation on the activity of *CbE1*Xyn43-l

To evaluate activity on xylose or arabinose substituents, 3^3^-α-l-arabinofuranosyl-xylotetraose (XA^3^XX), 2^3^- α-l-arabinofuranosyl-xylotriose (A^2^XX), and 2^3^,3^3^-di-α-l-arabinofuranosyl-xylotriose (A^2,3^XX) (Megazymes) were used as substrates. One millimolar of each substrate was dissolved in phosphate/Na buffer 50 mM (pH 7.0), and then 20 µM of *CbE1*Xyn43-l was added to the substrate and incubated overnight at 65°C in thermocycler (T-Gradient, Biometra). After the incubation, the temperature was increased to 100°C for 10 min to stop the enzymatic reaction, and finally, the samples were filtrated and analyzed by High-Performance Anion Exchange Chromatography with Pulse Amperiometric Detection (HPAEC-PAD) as described below.

### Xylooligosaccharides from arabino/glucuronoxylans analyzed by HPAEC-PAD

XO production by *CbE1*Xyn43-l was determined using HPAEC-PAD (Dionex, Sunnyvale, CA, USA). Hydrolysis of 1% (wt/vol) birchwood xylan, quinoa stalks glucuronoarabinoxylan, and wheat arabinoxylan was performed as described in the previous sections. The reactions were stopped by increasing the temperature of the reaction to 100°C for 10 min. Then, the samples were diluted 1:20 with ultra-pure water and analyzed using HPAEC-PAD according to the procedure described in Salas-Veizaga et al. (14) and Falck et al. (44). In short, a 250 mm × 4 mm i.d., 8.5 µm, CarboPac PA200 column and guard column, 50 mm × 4 mm, of the same material (Dionex) were used. The mobile phase (100 mM NaOH) was run at 0.5 mL min^−1^, and a gradient of sodium acetate was applied: 0–20 min of 10–160 mM and 20–25 min of 160–400 mM. Xylose (Sigma-Aldrich, Germany), xylobiose (X2), xylotriose (X3), xylotetraose (X4), xylopentaose (X5), and xylohexaose (X6) (Megazyme, Ireland) were used as standards in the range from 20 to 0.5 µM. All samples and controls were run in triplicates in all the experiments described in this section.

## Data Availability

The genome sequence was submitted to NCBI under accession number JAWONS000000000.1.
